# Assessment of Mitochondrial Respiration During Hypothermic Storage of Liver Biopsies Following Normothermic Machine Perfusion

**DOI:** 10.3389/ti.2024.12787

**Published:** 2024-05-23

**Authors:** Julia Hofmann, Alexander Kofler, Melanie Schartner, Madita L. Buch, Martin Hermann, Bettina Zelger, Dietmar Öfner, Rupert Oberhuber, Theresa Hautz, Stefan Schneeberger, Andras T. Meszaros

**Affiliations:** ^1^ OrganLife Laboratory, Department of Visceral, Transplant and Thoracic Surgery, Medical University of Innsbruck, Innsbruck, Austria; ^2^ Institute of Pathology, Neuropathology and Molecular Pathology, Medical University of Innsbruck, Innsbruck, Austria

**Keywords:** liver transplantation, mitochondria, normothermic machine perfusion, high-resolution respirometry, hypothermic storage

## Abstract

Organ quality can be assessed prior to transplantation, during normothermic machine perfusion (NMP) of the liver. Evaluation of mitochondrial function by high-resolution respirometry (HRR) may serve as a viability assessment concept in this setting. Freshly collected tissue is considered as optimal sample for HRR, but due to technical and personnel requirements, more flexible and schedulable measurements are needed. However, the impact of cold storage following NMP before processing biopsy samples for mitochondrial analysis remains unknown. We aimed at establishing an appropriate storage protocol of liver biopsies for HRR. Wedge biopsies of 5 human livers during NMP were obtained and assessed by HRR. Analysis was performed after 0, 4, 8, and 12 h of hypothermic storage (HTS) in HTK organ preservation solution at 4°C. With HTS up to 4 h, mitochondrial performance did not decrease in HTS samples compared with 0 h (OXPHOS, 44.62 [34.75–60.15] pmol·s^−1^·mg wet mass^−1^ vs*.* 43.73 [40.69–57.71], median [IQR], *p* > 0.999). However, at HTS beyond 4 h, mitochondrial respiration decreased. We conclude that HTS can be safely applied for extending the biopsy measurement window for up to 4 h to determine organ quality, but also that human liver respiration degrades beyond 4 h HTS following NMP.

## Graphical Abstract

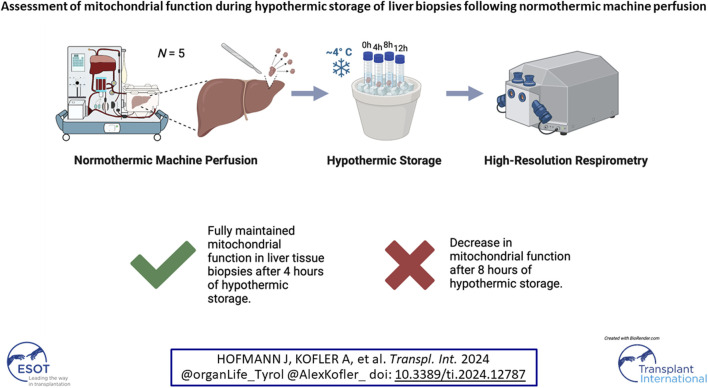

## Introduction

The serious shortage of donor organs is a daily challenge in liver transplantation (LT). To overcome this limitation and reduce waiting list mortality, extended criteria donor (ECD) organs are increasingly utilized for LT [1]. However, a substantial proportion of retrieved organs are not transplanted since they are considered inferior in their macroscopic appearance and expected function. Thus, it is essential to evaluate the quality of these grafts and predict the clinical outcome with the help of reliable biomarkers [2]. Machine perfusion is an emerging organ preservation strategy. Donor livers are perfused under normothermic conditions, which also allows for assessment of the organ function prior to transplantation. For this purpose, several biomarkers have been recently suggested. Transplant centers usually rely on a combination of different parameters for decision-making, including lactate clearance, level of perfusate transaminases, bile production and its composition. However, since robustness of those parameters with regard to their predictive capacity is limited, new biomarkers are under investigation [3–5]. Recently, our group demonstrated that mitochondrial bioenergetics assessed by high-resolution respirometry (HRR) in liver biopsies during the first 6 h of NMP predicts graft function after LT [6]. Different methods to analyze mitochondrial status are available, including monitoring free flavin mononucleotide [7–10], a mitochondrial damage parameter. In contrast, HRR allows for evaluation of the actual bioenergetic capacity by assessing mitochondrial respiration. Oxygen consumption of mitochondria is modulated by various substrates and inhibitors and is measured in real-time, thus enabling a comprehensive characterization of energy production, mitochondrial (dys) function and metabolic pathways.

In the above study, we performed HRR in tissue homogenates of freshly collected biopsies. However, this might be challenging in the clinical routine, especially in serial biopsies requiring immediate measurements, each taking approximately 2 h. To reduce this logistical and technical burden, extended hypothermic storage (HTS) would require little effort and ease the logistics. HRR analyses of tissue samples collected in the early phase of NMP could be performed in parallel, and the results would be available in a relevant period of the decision-making process for transplantation [11, 12]. The effect of cold ischemia on mitochondrial respiration has been extensively studied in endothelial cells [13], in heart [14], and in small samples of porcine livers [15]. However, no data on preservation protocols for liver biopsies taken during NMP exist. In this study we aimed to investigate the effect of HTS on mitochondrial respiration and coupling control in clinical liver NMP.

## Material and Methods

### Study Design and Ethics

Tissue samples of liver allografts (*N* = 5) subjected to NMP (Metra^®^, OrganOx Limited, Oxford, United Kingdom) prior to transplantation were included in this single center experimental study between January and August 2023. The study was approved by the institutional ethics committee (Ethics Commission of the Medical University of Innsbruck, EK Nr. 1175/2018). NMP was performed in accordance with our center’s protocols as described by Cardini *et al.* [16, 17]. After organ acceptance and indication for NMP subjection, livers were included in the study on a rolling-basis when (i) logistics could be arranged and (ii) repetitive high-resolution respirometry measurements during the next 12 h could be ensured. At the time point of inclusion, the performance of the livers during NMP was still under investigation. Wedge biopsies (20 mg ± 2 mg wet mass) were taken during clinical NMP and divided into 4 approximately equal parts (∼5 mg each). The biopsy samples were then directly placed in ice-cold HTK solution (Custodiol^®^, Dr. Franz Köhler Chemie GmbH, Bensheim, Germany) and transferred to the laboratory at 4°C on ice for further processing. The first biopsy was immediately analyzed for baseline mitochondrial respiratory function values. The further samples were stored at 4°C on ice and analyzed after 4, 8, or 12 h of HTS.

### High-Resolution Respirometry (HRR)

Mitochondrial respiration was assessed by HRR (O2k, Oroboros Instruments, Innsbruck, Austria) with devices equipped with two identical 0.5 mL measurement chambers at 37°C and under continuous stirring at 750 rpm [18]. Measurements were preceded by air-calibration of the devices using the mitochondrial respiration medium MiR05 (MiR05-Kit, Oroboros Instruments, Innsbruck, Austria) containing 0.5 mM EGTA, 3 mM MgCl_2_ • 6 H_2_O, 60 mM lactobionic acid, 20 mM taurine, 10 mM KH_2_PO_4_, 20 mM HEPES, 110 mM D-sucrose, 1 g·L^−1^ essentially fatty acid free bovine serum albumin). Liver wedge biopsies were weighted in a 4°C environment, and subsequently homogenized in 4°C MiR05 using a PBI-Shredder O2k-Set (Oroboros Instruments, Innsbruck, Austria), to disrupt cellular structures and extract mitochondria. The crude tissue homogenate, containing freely accessible mitochondria, was adjusted to a final concentration of 1 mg wet mass·mL^−1^, and 500 µL was transferred into the O2k chambers. Reagents were added using Hamilton glass microsyringes (Hamilton Bonaduz AG, Bonaduz, Switzerland) following pre-defined Substrate-Uncoupler-Inhibitor Titration (SUIT) protocols. All measurements were performed in technical duplicates [6]. Oxygen concentration and flux were recorded at an interval of 2 s using DatLab 7 software (Datlab 7.4, Oroboros Instruments, Innsbruck, Austria).

### Substrate-Uncoupler-Inhibitor Titration (SUIT) Protocols

**FIGURE 1:**
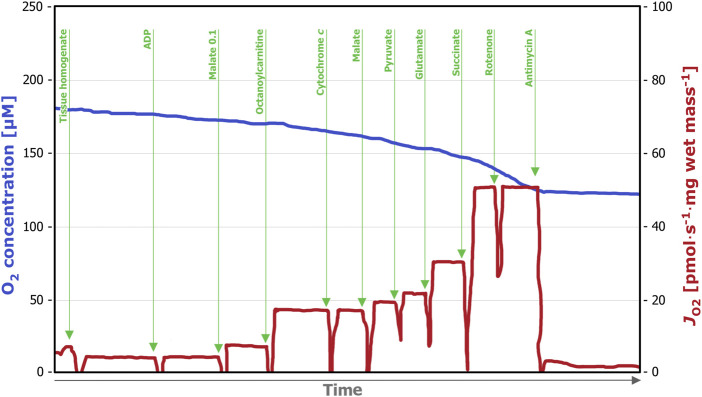
Representative trace of a high-resolution respirometry measurement applying the SUIT (i) protocol. After addition of the tissue homogenate (0.5 mL), residual oxygen consumption (ROX) was assessed in the absence of fuel substrates and adenosine diphosphate (ADP). Next, ADP (5 µM) was added, followed by malate (0.1 mM) and octanoylcarnitine (0.5 mM), which allows for assessment of the F_*P*_ OXPHOS capacity. Integrity of the outer mitochondrial membrane was tested by titration of cytochrome *c* (10 µM). The subsequent titration of N_*P*_-linked substrates (2 mM malate, 5 mM pyruvate and 10 mM glutamate) enables evaluation of combined F_*P*_- and N_*P*_ -linked OXPHOS capacity. Next, F_*P*_-, N_*P*_, and S_*P*_-linked OXPHOS capacity was measured by the addition of succinate (10 mM), followed by the titration of Complex I inhibitor rotenone (0.5 µM), which allows the measurement of S_*P*_-linked OXPHOS capacity only. Lastly, the Complex III inhibitor antimycin A (2.5 µM) was added, which allows for evaluation of ROX. OXPHOS, oxidative phosphorylation; F_*P*_, fatty acid oxidation-linked pathway; N_*P*_, nicotinamide adenine dinucleotide-linked pathway; S_*P*_, succinate-linked pathway; ROX, residual oxygen consumption.

We applied two different SUIT protocols. SUIT(i), corresponding SUIT-025 in the online protocol library Bioblast^1^, (Figure 1) allows for evaluation of the substrate pathway control in the OXPHOS state. After addition of ADP in saturating concentrations (5 mM), the substrates for the fatty acid oxidation-linked pathway (F_*P*_), malate (0.1 mM) and octanoylcarnitine (0.5 mM) were titrated, followed by cytochrome *c* (10 µM) for the evaluation of the integrity of the mitochondrial outer membrane. Next, substrates for the NADH-linked pathway (N_*P*_); pyruvate (5 mM), malate (2 mM) and glutamate (10 mM) were added. The overall capacity of convergent electron transfer pathways was determined after succinate addition (10 mM) (FNS_*P*_). For determination of the succinate-linked pathway alone, (S_*P*_) rotenone (0.5 µM) was titrated. Finally, to inhibit Complex III and determine residual oxygen consumption (ROX), antimycin A (2.5 µM) was added.

**FIGURE 2:**
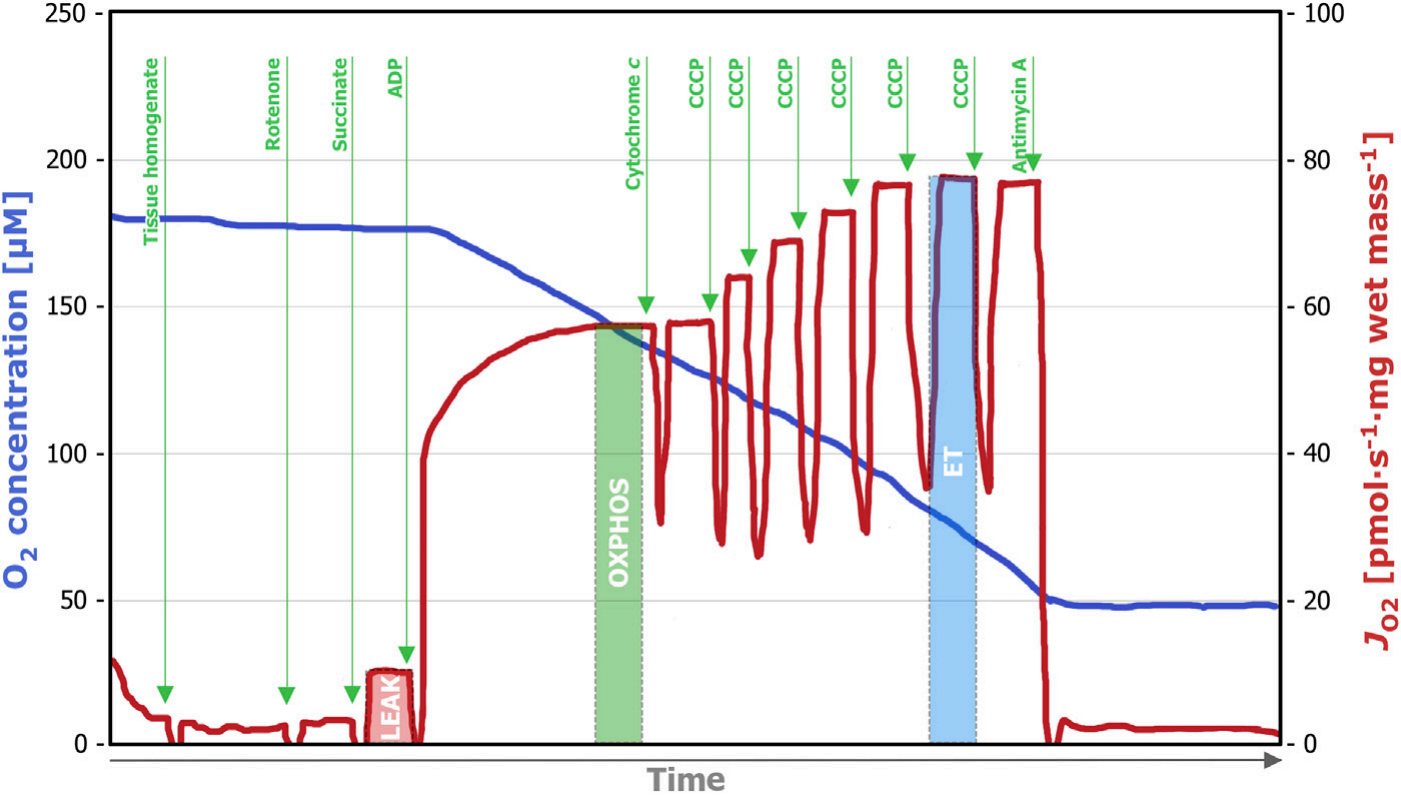
Representative trace of a high-resolution respirometry measurement applying the SUIT (ii) protocol. After addition of the tissue homogenate (0.5 mL), residual oxygen consumption (ROX) was assessed in the absence of fuel substrates and adenosine diphosphate (ADP). Titration of rotenone (0.5 µM) inhibits Complex I and after the subsequent addition of succinate (10 mM), LEAK respiration could be measured. The addition of ADP (5 mM) enables S_*P*_-linked OXPHOS capacity assessment and with the subsequent titration of cytochrome *c* (10 µM) the integrity of the outer mitochondrial membrane can be evaluated. To assess the electron transfer (ET) capacity, step-wise titration (0.5 µM per step) of CCCP until maximum respiration was performed. Lastly, the Complex III inhibitor antimycin A (2.5 µM) was added, inducing ROX. OXPHOS, oxidative phosphorylation; S_*P*_, succinate-linked pathway; CCCP, carbonyl cyanide p-trifluoromethoxyphenyl hydrazone.

SUIT(ii), corresponding SUIT-006 in the online protocol library Bioblast^2^ (Figure 2) provides detailed information on the S_*P*_-linked mitochondrial respiration. Rotenone (0.5 µM) was added for inhibition of the mitochondrial Complex I, which was followed by succinate (10 mM) titration. Next, ADP (5 mM) for evaluation of the OXPHOS capacity was added followed by cytochrome *c* (10 µM) for the evaluation of the integrity of the mitochondrial outer membrane. Carbonyl cyanide p-trifluoromethoxyphenyl hydrazone (CCCP) was titrated stepwise (0.5 µM per step) to assess non-coupled respiration. Finally, antimycin A (2.5 µM) was added to achieve ROX.

### Mitochondrial Coupling Control States

Respiration rates were expressed as O_2_ flux per wet mass tissue [pmol·O_2_·s^−1^·mg^−1^]. In addition, respiratory capacities were normalized to an internal reference state for each measurement to determine flux control ratios (*FCR*) for evaluation of SUIT(i). For SUIT(ii), the coupling states LEAK (*L*), OXPHOS (*P*), OXPHOS(*c*) (*P*_*c*_), and Electron Transfer capacity (*ET*) were evaluated. Based on the above, the following control efficiencies were calculated: *P-L* control efficiency (1-*L*/*P*), to evaluate the efficiency of the ATP production within S_*P*_, and cytochrome *c* control efficiency (1-*P*/*P*_*c*_) to evaluate the damage to the mitochondrial outer membrane [17, 19].

### Real-Time Confocal Microscopy (RTCM)

Tissue integrity and cell viability of the obtained biopsies were assessed by real-time confocal microscopy (RTCM) as previously described [20, 21]. Briefly, biopsies were incubated with the fluorescent stains Hoechst 33,342 (blue, staining all nuclei, Molecular Probes; final concentration 5 μg·mL^−1^), propidium iodide (PI; red, staining the nuclei of dead cells, Molecular Probes; final concentration 500 nM), and wheat germ agglutinin (WGA; green, staining of cell morphology, Molecular Probes, Eugene, OR, United States; 5 μg·mL^−1^ final concentration) at room temperature for 15 min. Image acquisition was performed with an UltraVIEW VoX spinning disk confocal system (Perkin Elmer, Wellesley, MA) connected to a Zeiss AxioObserver Z1 microscope (Zeiss, Oberkochen, Germany). Quantification relied on a semi-quantitative scoring system (0-1-2): 0—high number of viable cells, 1—an equal count of viable and dead cells, and 2—low number of viable cells [6].

### Histopathology

Liver biopsies were fixed in 10% formalin for 24 h, then embedded in paraffin and cut in 3 µM sections. Hematoxylin and eosin (H&E) staining was performed following standard protocols to assess necrosis, inflammation, steatosis, fibrosis, and vascular changes. For scoring a modified Suzuki score was applied [6].

### Statistical Analysis

Shapiro-Wilk test was employed to examine the normality of the data. Continuous variables are represented as median [interquartile range (IQR)]. Categorial variables are expressed as percentage (%). To assess differences compared to baseline values (0 h sample), Friedman’s test with a post-hoc comparison to examine specific pairwise differences by the Dunn’s method was performed. Statistical analysis was performed using GraphPad Prism 10 and a *p*-value of <0.05 was considered statistically significant.

## Results

### Donor and Preservation Characteristics

5 human livers undergoing NMP were included in this study. Four grafts (80%) originated from donors after brain death (DBD). The median age of the donors was 72 [54–78] years, with a median body mass index of 29 [23–33] kg·m^−2^. The median donor risk index (DRI) was 2.04 [1.94–2.37], and 80% of organs were procured from ECD. One liver was found suitable for transplantation after NMP, while four livers deemed unsuitable and had to be declined. The individual donor and preservation characteristics, viability parameters and reasons for decline can be found in Table 1.

**TABLE 1:** Donor and preservation characteristics.

Donor characteristics	Liver 1	Liver 2	Liver 3	Liver 4	Liver 5
Age (years)	65	43	72	79	77
Sex	M	M	M	M	M
Body mass index (kg·m^−2^)	29	23	37	29	23
Cause of death	CVA	CVA	CVA	CVA	CVA
Donor type	DBD	DCD	DBD	DBD	DBD
ECD status	no	yes	yes	yes	yes
DRI	1.92	2.43	2.04	2.31	1.96
Steatosis^a^	mild	no	mild	no	no
Fibrosis^a^	mild	no	no	no	no
Hepatitis^a^	minimal	no	no	no	no

Preservation	Liver 1	Liver 2	Liver 3	Liver 4	Liver 5
CIT (min)	538	599	492	306	365
Functional WIT^b^ (DCD; min)		18			
Lactate ≤2.5 mmol⋅L^−1^ after 2 h NMP	yes	yes	yes	yes	no
AST peak (NMP) (U⋅L^−1^)	2,417	11,221	1,048	2,159	12,800
ALT peak (NMP) (U⋅L^−1^)	2,588	3,327	909	1,515	11,655
LDH peak (NMP) (U⋅L^−1^)	5,596	16,339	3,036	3,270	25,507
Bile production during NMP	no	yes	yes	yes	yes
Reason for decline	pH instable, no bile production	Inhomogeneous perfusion after complex arterial reconstruction	Transplanted	Recipient tested positive for COVID-19, liver was reallocated	Late lactate clearance, cholestasis, high LDH

^a^ Assessed in frozen tissue sections.

^b^ In the context of DCD donors, functional warm ischemia time refers to the interval starting when the mean arterial pressure falls below 50 mmHg or arterial saturation drops below 80%, and ending when cold perfusion starts.

CVA, cerebrovascular accident; ECD, extended criteria donor; DBD, donation after brain death; DCD, donation after circulatory death; DRI, donor risk index; CIT, cold ischemic time; WIT, warm ischemic time; NMP, normothermic machine perfusion; AST, aspartate aminotransferase; ALT, alanine aminotransferase; LDH, lactate dehydrogenase.

### High-Resolution Respirometry for Assessment of Hypothermically Stored Liver Biopsies

**FIGURE 3:**
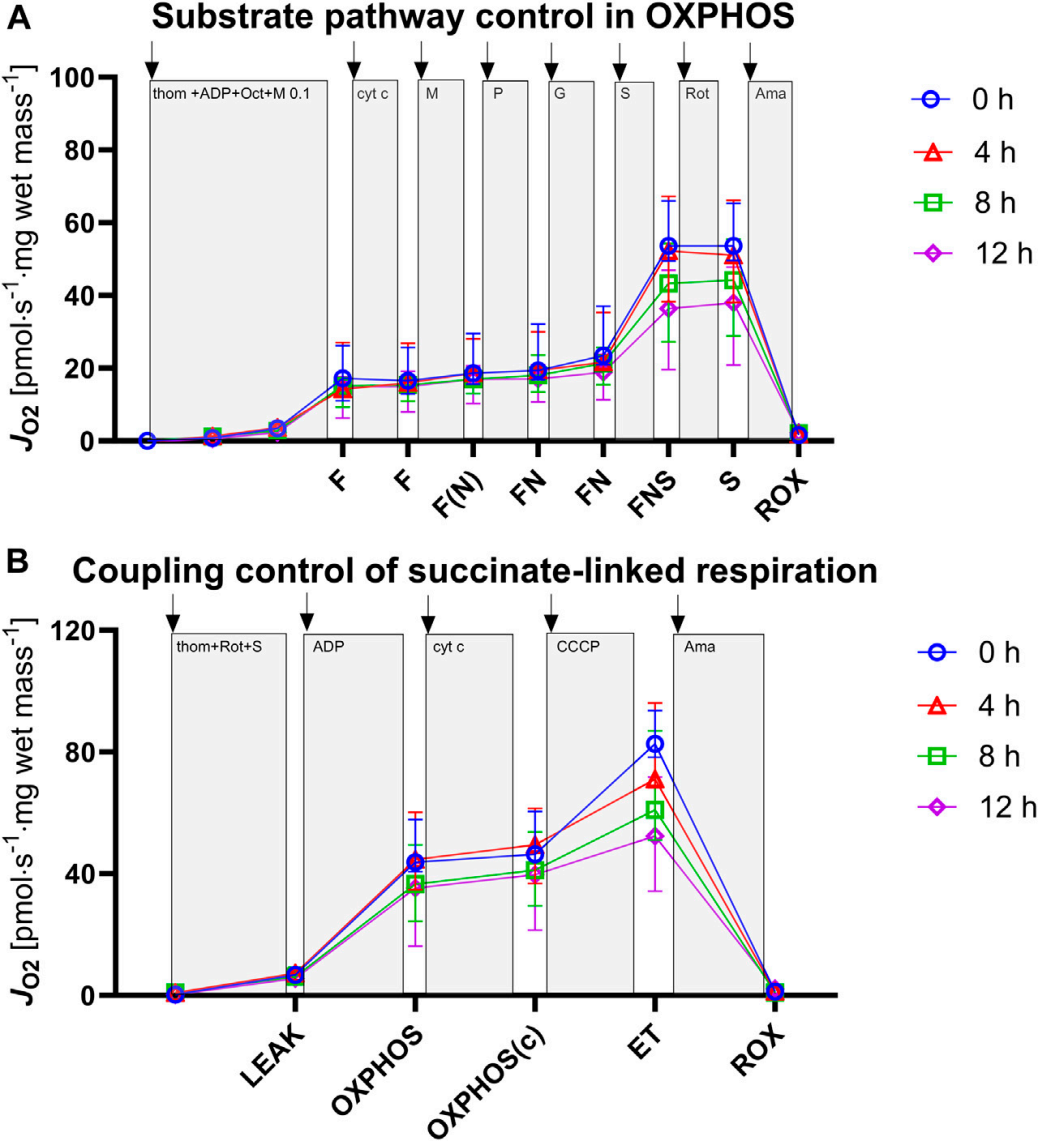
Assessment of mitochondrial respiration in tissue biopsies after 0, 4, 8, and 12 h of hypothermic storage. **(A)** ROX was measured after addition of tissue homogenate and was followed by titration of ADP to evaluate OXPHOS capacities for the F, FN and FNS pathway states. **(B)** ROX was measured after addition of tissue homogenate followed by Complex I inhibition and succinate titration to evaluate S-linked LEAK respiration. ADP and cytochrome *c* addition allowed for measurement of OXPHOS capacity and OXPHOS (c) capacity, respectively. Uncoupler titrations were performed to analyze ET capacity. ADP, adenosine diphosphate; F, fatty acid oxidation; FN, fatty acid oxidation and NADH; FNS, fatty acid oxidation-NADH-succinate; OXPHOS, oxidative phosphorylation; ET, electron transfer; ROX, residual oxygen consumption; thom, tissue homogenate; Oct, octanoylcarnitine; M, malate; P, pyruvate; G, glutamate; S, succinate; Rot, rotenone; Ama, antimycin A**;** cyt c, cytochrome *c*; CCCP, Carbonyl cyanide p-trifluoromethoxyphenyl hydrazone. Data are represented as median and interquartile range.

With the progression of hypothermic storage, gradual, time-dependent changes in mitochondrial respiration were observed (Figure 3).

### Characterization of Pathway Contribution

**FIGURE 4:**
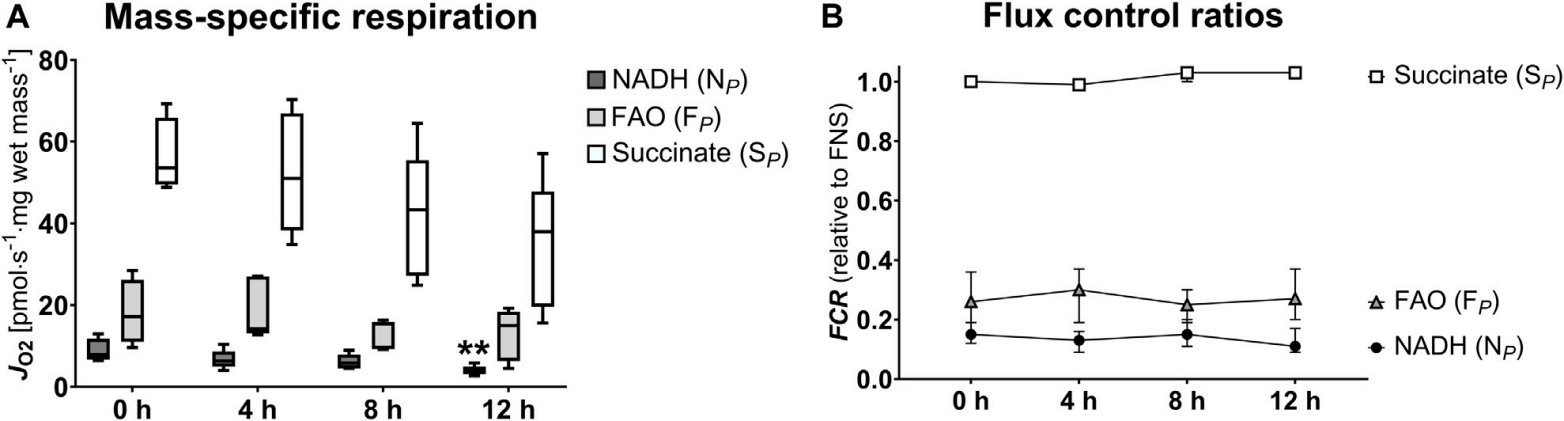
Pathway-specific mitochondrial respiration **(A)** and flux control ratios **(B)**. NADH, nicotinamide adenine dinucleotide hydrogen; FAO, fatty acid oxidation; S, succinate; FCR, flux control ratio. **(A)**: Data are represented as median, interquartile range and min-max. **(B)**: Data are represented as median and interquartile range. Friedman’s test with Dunn’s multiple comparisons was applied to assess differences compared to 0 h samples. **p < 0.01.

We investigated the NADH- (N_*P*_), FAO- (F_*P*_), and succinate-linked (S_*P*_) oxidative phosphorylation (Figure 4; Table 2). Respiration was highest for S_*P*_ and lower for N_*P*_ and F_*P*_. Concerning S_*P*_, fresh and 4 h HTS measurements revealed similar respiration rates (53.59 [49.51–65.87] pmol·s^−1^·mg wet mass^−1^ vs*.* 51.01 [38.31–66.91] pmol·s^−1^·mg wet mass^−1^, *p* > 0.999), while after 8 h HTS a marked decrease in respiration was observed (43.32 [27.22–55.48] pmol·s^−1^·mg wet mass^−1^, *p* = 0.811). At 12 h, the respiration was even lower: (37.99 [19.61–47.79] pmol·s^−1^·mg wet mass^−1^, *p* = 0.06). In line with this, N_*P*_ and F_*P*_ remained stable for 4 h of HTS and declined after 8 h: N_*P*_ decreased to 3.89 [3.10–4.94] pmol·s^−1^·mg wet mass^−1^ after 12 h (*p* = 0.006). Furthermore, the flux control ratios for each pathway were calculated, to take advantage of the internal normalization relative to the maximal convergent electron pathway respiration (Figure 4; Table 2). In the present cohort, in fresh biopsies FCRs of 0.27 and 0.14 were found for F_*P*_ and N_*P*_, respectively, which also remained constant over storage time. For S_*P*_ a FCR of 1.0 was observed for fresh biopsies and all analyzed HTS biopsies, which indicated that succinate alone is sufficient to saturate OXPHOS capacity.

**TABLE 2:** Pathway-specific oxidative phosphorylation.

Mitochondrial respiration (pmol·s^−1^·mg wet mass^−1^)	0 h	4 h	*p*-value	8 h	*p*-value	12 h	*p*-value
NADH (N_*P*_)	7.84 [6.65–11.78]	6.30 [4.95–8.66]	0.981	5.86 [4.52–7.88]	>0.999	3.89 [3.10–4.94]	0.006
FAO (F_*P*_)	17.20 [11.00–26.18]	14.17 [13.08–27.03]	>0.999	15.40 [9.26–15.89]	0.534	14.99 [6.31–18.38]	0.334
Succinate (S_*P*_)	53.59 [49.51–65.87]	51.01 [38.31–66.91]	>0.999	43.32 [27.22–55.48]	0.811	37.99 [19.61–47.79]	0.06
**FCR (relative to *FNS*)**	**0 h**	**4 h**	***p*-value**	**8 h**	***p*-value**	**12 h**	***p*-value**
NADH (N_*P*_)	0.15 [0.13–0.18]	0.13 [0.10–0.15]	0.662	0.15 [0.12–0.19]	>0.999	0.11 [0.10–0.17]	>0.999
FAO (F_*P*_)	0.26 [0.17–0.35]	0.30 [0.21–0.34]	0.534	0.25 [0.22–0.30]	>0.999	0.27 [0.23–0.35]	0.259
Succinate (S_*P*_)	1.00 [0.99–1.00]	0.99 [0.99–0.99]	>0.999	1.03 [1.01–1.08]	0.199	1.03 [1.02–1.07]	0.150

NADH, nicotinamide adenine dinucleotide hydrogen; FAO, fatty acid oxidation; FCR, flux control ratio; FNS, fatty acid oxidation-NADH-succinate. Data are represented as median, interquartile range and min-max. Friedman’s test with Dunn’s multiple comparisons was applied to assess differences compared to 0 h samples.

### Succinate-Linked Mitochondrial Respiration

**FIGURE 5:**
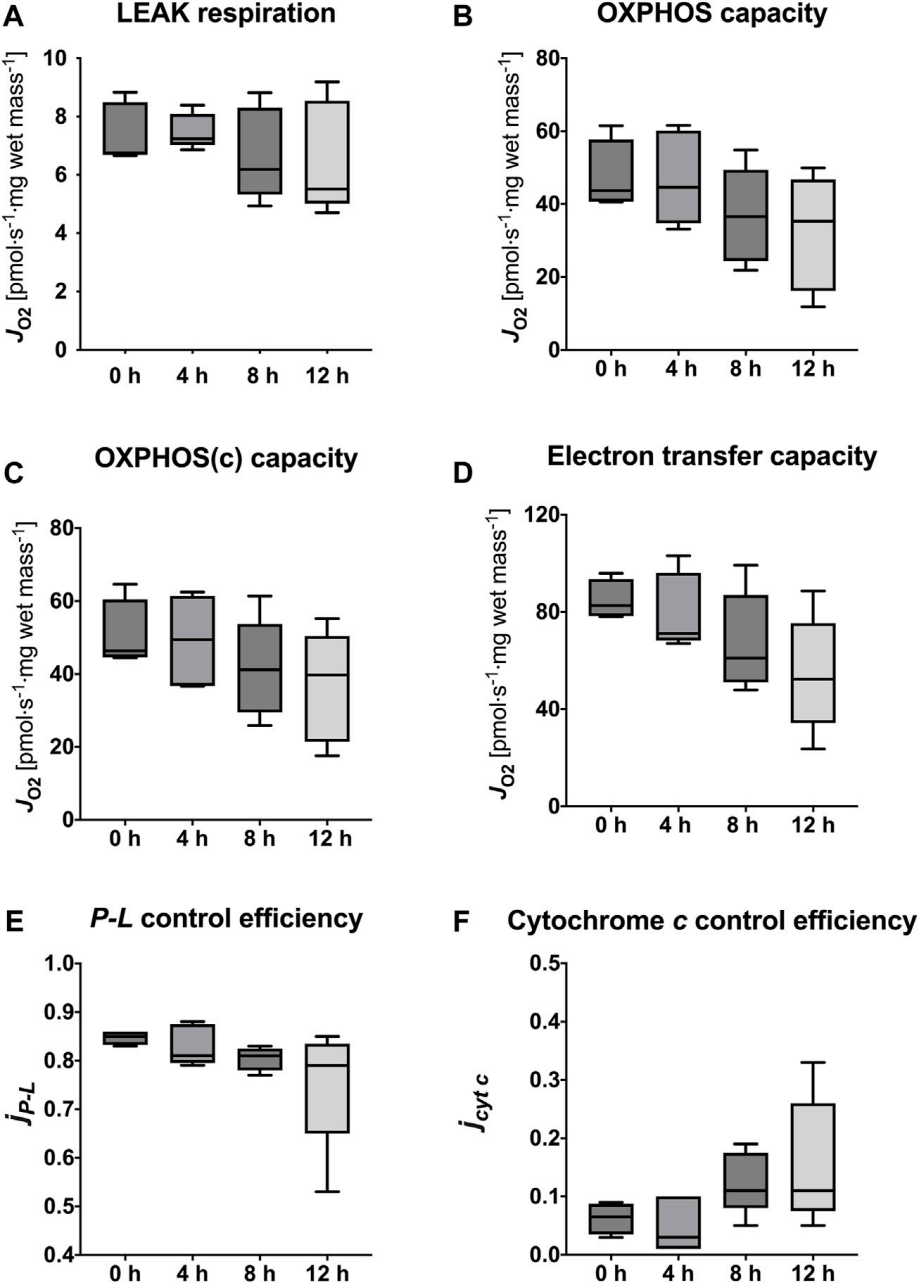
Mitochondrial respiratory states and coupling control efficiencies. LEAK respiration **(A)**, OXPHOS capacity **(B)**, OXPHOS(c) capacity **(C)**, Electron transfer capacity **(D)**, *P-L* control efficiency **(E)** to evaluate efficiency of ATP production, and cytochrome *c* control efficiency **(F)** to evaluate integrity of the outer mitochondrial membrane. Data are represented as median, interquartile range and min-max. Friedman’s test with Dunn’s multiple comparisons was applied to assess differences compared to 0 h samples. No significant differences were found (*p* < 0.05).

The succinate-linked respiration was analyzed in greater detail as it relates to the prominent substrate pathway in the present and previous studies (Table 3; Figure 5). Measurement of LEAK respiration, representing the non-phosphorylating component of mitochondrial respiration, yielded similar results between fresh tissue biopsies and those measured after 4 h HTS (6.75 [6.69–8.50] pmol·s^−1^·mg wet mass^−1^ vs*.* 7.24 [7.03–8.09] pmol·s^−1^·mg wet mass^-1^, *p* > 0.999). In line with this, OXPHOS capacity, measured with saturating substrate, ADP, and inorganic phosphate concentrations remained stable for 4 h HTS (fresh 43.73 [40.69–57.71] pmol·s^−1^·mg wet mass^−1^ vs*.* 4 h HTS 44.62 [34.75–60.15] pmol·s^−1^·mg wet mass^−1^, *p* > 0.999). The same pattern was also observed for the electron transfer capacity in the non-coupled state (fresh 82.64 [78.22–93.51] pmol·s^−1^·mg wet mass^−1^ vs*.* 4 h HTS 71.09 [68.24–96.07] pmol·s^−1^·mg wet mass^−1^, *p* > 0.999). All assessed parameters showed decrease after 8 h HTS and beyond. However, significance was not reached.

**TABLE 3:** Mitochondrial respiratory capacities of the S-pathway.

Coupling states (pmol·s^−1^·mg wet mass^−1^)	0 h	4 h	*p*-value	8 h	*p*-value	12 h	*p*-value
LEAK	6.75 [6.69–8.50]	7.24 [7.03–8.09]	>0.999	6.19 [5.33–8.30]	>0.999	5.51 [5.02–8.54]	>0.999
OXPHOS	43.73 [40.69–57.71	44.62 [34.75–60.15]	>0.999	36.56 [24.38–49.41]	0.811	35.29 [16.18–46.77]	0.199
OXPHOS(c)	46.31 [44.55–60.41	49.42 [36.75–61.42]	>0.999	41.17 [29.47–53.69]	0.811	39.71 [21.42–50.43]	0.199
ET	82.64 [78.22–93.51]	71.09 [68.24–96.07]	>0.999	60.95 [51.07–86.91]	0.811	52.33 [34.26–75.40]	0.06

OXPHOS, oxidative phosphorylation; OXPHOS(c), OXPHOS capacity after cytochrome *c* addition; ET, electron transfer. Data are represented as median, interquartile range and min-max. Friedman’s test with Dunn’s multiple comparisons was applied to assess differences compared to 0 h samples.

### Coupling Control Efficiencies

The calculated mitochondrial coupling control efficiencies of the succinate-pathway did not show significant alterations during HTS up to 12 h compared to fresh biopsies (Table 4). Calculation of the *P-L* control efficiency allows for evaluation of the efficiency of ATP production. It was sufficiently high in fresh biopsies (0.85 [0.83–0.86]) but a trend towards lower values has been observed after storage for 12 h (0.79 [0.65–0.84], *p* = 0.112). Furthermore, cytochrome *c* control efficiency, which reflects the integrity of the mitochondrial outer membrane, revealed a slight increase at 8 h and 12 h HTS, indicating higher membrane permeability (*p* = 0.199 and *p* = 0.112) (Figure 5).

**TABLE 4:** Coupling control efficiencies of the succinate-linked pathway.

Coupling control efficiencies	0 h	4 h	*p*-value	8 h	*p*-value	12 h	*p*-value
*P-L* control efficiency	0.85 [0.83–0.86]	0.81 [0.80–0.88]	>0.999	0.81 [0.78–0.83]	0.150	0.79 [0.65–0.84]	0.112
Cytochrome *c* control efficiency	0.07 [0.04–0.09]	0.03 [0.01–0.10]	>0.999	0.11 [0.08–0.18]	0.199	0.11 [0.08–0.26]	0.112

Data are represented as median, interquartile range and min-max. Friedman’s test with Dunn’s multiple comparisons was applied to assess differences compared to 0 h samples.

### Real-Time Confocal Microscopy and Conventional Histology

**FIGURE 6:**
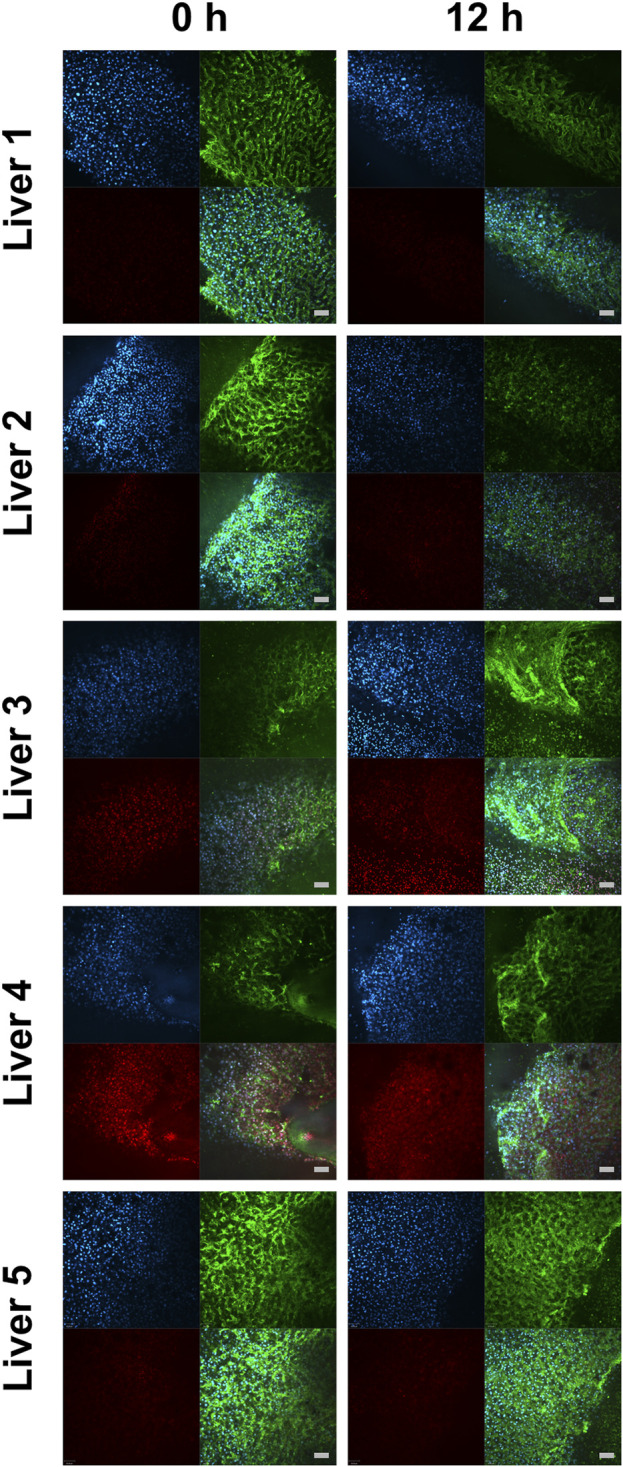
Real-time confocal microscopy of liver biopsies immediately after normothermic machine perfusion and after 12 h hypothermic storage. Images from real-time confocal microscopy portray cells stained with Hoechst 33,342 (in blue, detection of all nuclei), PI (in red, detection of dead cell’s nuclei), WGA (in green, cell morphology), and the merged view. The scale bars denote 100 μm.

In contrast to mitochondrial respiration, tissue integrity and cell viability could be preserved for up to 12 h of HTS, as demonstrated by RTCM analysis (Figure 6). The semiquantitative RTCM score revealed no changes during the storage period (data not shown). The microscopic evaluation of H&E sections also showed no differences regarding histopathology when comparing fresh biopsies with 12 h HTS biopsies (Supplementary Figure S1). This also underlines the sensitivity of HRR in comparison to other assessment methods during cold storage.

## Discussion

Herein we investigated the feasibility of prolonged hypothermic storage of tissue biopsies taken during NMP for later HRR analysis. Testing of mitochondrial respiration in livers undergoing NMP can provide information on organ function and displays a reliable biomarker for decision-making [6, 17]. The HRR parameters cytochrome *c* control efficiency, *P-L* control efficiency, and LEAK respiration of the succinate-linked pathway correlated with the clinical outcome after transplantation in a previous clinical study. While HRR is a sensitive method, it requires technical proficiency of the operators and measurement immediately after sample collection. Enabling postponement of mitochondrial respiratory assessment would allow to analyze more biopsies and reduce the burden on personnel. This aspect is particularly relevant in scenarios where a higher number of biopsies need to be assessed for a comprehensive understanding of organ viability.

Our findings shed light on the feasibility of preservation of mitochondrial function in hypothermically stored liver biopsies. It indicates that prolonged periods of cold storage following liver NMP might be harmful, while shorter storage times of up 4 h may be acceptable. Our results demonstrated that mitochondrial respiration remained stable within this timeframe, and thus reliable results on mitochondrial function can be obtained after this storage period. This could guide decision-making processes pertaining to organ acceptance or utilization. Interestingly, beyond the 4-h mark mitochondrial respiration and efficiency tend to decrease, while the mitochondrial outer membrane damage increases. This contrasts earlier findings of our department [15], where the preservation of OXPHOS capacity and cytochrome *c* control efficiency during 48 h of hypothermic storage in porcine livers were analyzed. The first key difference between these experiments is that livers analyzed in the present study have variable and notably longer cold ischemic times and have undergone reperfusion in the form of NMP, while in the previous study no machine perfusion was applied. This finding is in line with a recent study of Horváth *et al.* [22]. They investigated the effect of prolonged CIT and subsequent normothermic isolated perfusion on mitochondrial function in DCD rat livers. Mitochondrial respiration was decreased after 24 h of additional CIT compared to the control group, although in their setting no oxygen carrier was applied, which negatively affects mitochondrial metabolism under normothermic conditions [23]. Furthermore, it has already been shown in small animal models that NMP leads to altered metabolomic signatures in liver tissue which affect mitochondrial biochemical pathways, such as reduced succinate levels. This holds also potential for using different perfusion models and various optimizing supplements to positively influence liver metabolism during NMP [24, 25]. The second major difference is the quality of the organs. Healthy porcine livers without preexisting damage seem to be less sensitive to mitochondrial damage when compared to human livers coming from aged, ECD and/or DCD donors. While in our recent study no decrease in mitochondrial respiration during the first 6 h of NMP was found in the overall cohort, individual changes in some livers could be demonstrated.

While the decreased respiration in biopsies at both the 8 h and 12 h HTS preservation did not reach statistical significance, the trends are clear, and this nonetheless underscores a potential threat of prolonged HTS following NMP. The decrease in mitochondrial function after reaching 8 h of HTS preservation could be attributed to several underlying factors such as the accumulation of metabolic byproducts, disruption of cellular homeostasis and ion gradients, oxidative stress, and consequent alterations in mitochondrial membrane integrity [1, 11]. This level of degradation at 8 h may warrant further exploration to better understand the mechanisms involved and potentially improve preservation methods. It is noteworthy that both the RTCM and the histopathological examinations did not show any appreciable differences between samples at 0 and 12 h of HTS.

Our study acknowledges certain limitations that pertain to both HRR and the organ quality. The reproducibility of HRR measurements can be influenced by sample variability, experimental conditions, technical factors, and from the measurement process itself, often influenced by the individual conducting the measurements. This can impact accuracy of the results, highlighting the importance of standardized protocols, training, quality control, and minimizing inter-operator variability [26]. In addition, the relatively small sample number of *N* = 5 livers and single-center design, indicate a possible frailty, which needs to be proven by further centers. While the stability of mitochondrial respiration up to 4 h upon sample collection in an NMP setting could be demonstrated, the actual clinical outcome could not be predicted in the 4 h samples as 4 of 5 livers have been rejected during NMP, based on conventional organ quality criteria.

In summary, this study accentuates the value of HRR in assessing the bioenergetic efficacy of livers during cold storage and the dynamics of mitochondrial stability and function during static cold storage following NMP. HTS of tissue biopsies of livers undergoing NMP can be performed for no longer than 4 h. While further research is needed, our findings underscore the potential of this simple method as a tool for informed decision-making during evaluation for organ transplantation, with the added benefit of easing the logistic challenges of HRR and facilitating concurrent assessment of a larger number of biopsies.

## Figures and Tables

**Table:** 

**ATP**	adenosine triphosphate
**CCCP**	Carbonyl cyanide p-trifluoromethoxyphenyl hydrazone
**CIT**	cold ischemia time
**CVA**	cerebrovascular accident
**DBD**	donation after brain death
**DCD**	donation after circulatory death
**DRI**	donor risk index
**ECD**	extended criteria donor
**ET**	electron transfer
**F**	FAO-linked pathway
**FAO**	fatty acid oxidation
**FCR**	flux control ratio
**FMN**	flavin mononucleotide
**HRR**	high-resolution respirometry
**HTS**	hypothermic storage
**IQR**	interquartile range
**L**	LEAK respiration
**LEAK**	the state of non-phosphorylating resting respiration in the absence of ADP
**LT**	liver transplantation
**N**	NADH-linked pathway
**NADH**	nicotinamide adenine dinucleotide hydrogen
**NMP**	normothermic machine perfusion
**OXPHOS**	oxidative phosphorylation
**P**	OXPHOS: Respiratory capacity of mitochondria in the ADP-activated state of oxidative phosphorylation, at saturating concentrations of ADP and inorganic phosphate, oxygen, and defined substrates
**PI**	propidium iodide
**ROX**	residual oxygen consumption
**RTCM**	real-time confocal microscopy
**S**	succinate-linked pathway
**SUIT**	Substrate-Uncoupler-Inhibitor Titration
**WGA**	wheat germ agglutinin

## Data Availability

The raw data supporting the conclusion of this article will be made available by the corresponding author upon request.

## Footnotes

1
https://wiki.oroboros.at/index.php/SUIT-025

2
https://wiki.oroboros.at/index.php/SUIT-006
