# Supplementary material for: Assessment of Mitochondrial Respiration During Hypothermic Storage of Liver Biopsies Following Normothermic Machine Perfusion
Source: Transpl Int. 2024 May 23;37:12787. doi: 10.3389/ti.2024.12787 (PMC11153658; doi:10.3389/ti.2024.12787)
Supplement: Supplementary file 1 [file DataSheet1.docx]

***Supplementary Material***

**Assessment of mitochondrial function during hypothermic storage of liver biopsies following normothermic machine perfusion**

Julia Hofmann^1†^, Alexander Kofler^1†^, Melanie Schartner^1^, Madita L. Buch^1^, Martin Hermann^1^, Bettina Zelger^2^, Dietmar Öfner^1^, Rupert Oberhuber^1^, Theresa Hautz^1^, Stefan Schneeberger^1^, Andras T. Meszaros^1*^

^†^ These authors contributed equally to this work and share first authorship

***Correspondence:** Andras T. Meszaros: andras.meszaros@i-med.ac.at

**Supplementary Figures**

**
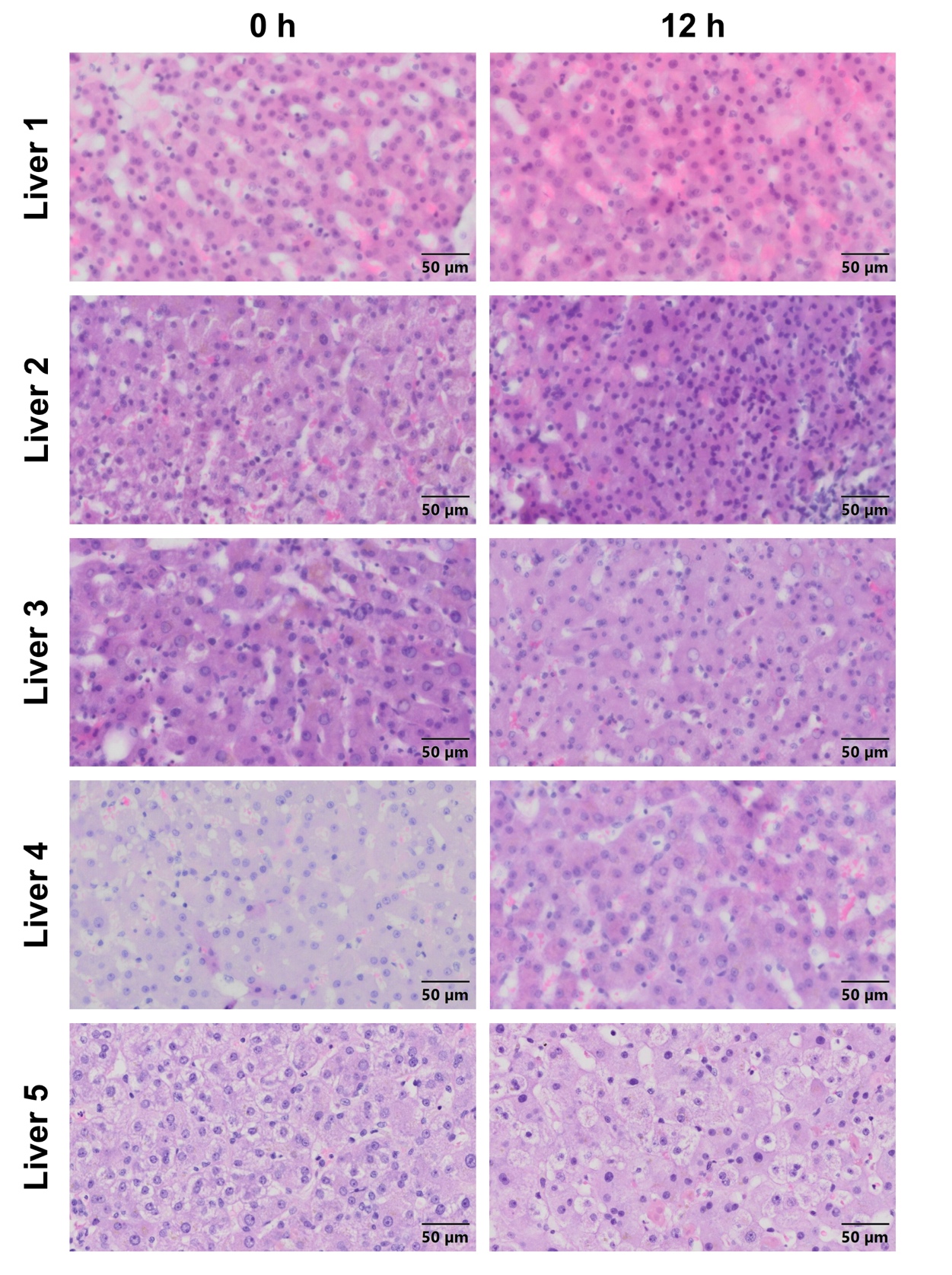
**

**Supplementary Figure 1: Photomicrographs of tissue biopsies post hematoxylin and eosin staining.** Liver tissue biopsies exhibited no histopathological changes during hypothermic storage.
